# Macrodomain ADP-ribosylhydrolase and the pathogenesis of infectious diseases

**DOI:** 10.1371/journal.ppat.1006864

**Published:** 2018-03-22

**Authors:** Anthony K. L. Leung, Robert Lyle McPherson, Diane E. Griffin

**Affiliations:** 1 Department of Biochemistry and Molecular Biology, Bloomberg School of Public Health, Johns Hopkins University, Baltimore, Maryland, United States of America; 2 Department of Oncology, School of Medicine, Johns Hopkins University, Baltimore, Maryland, United States of America; 3 W. Harry Feinstone Department of Molecular Microbiology and Immunology, Bloomberg School of Public Health, Johns Hopkins University, Baltimore, Maryland, United States of America; University of Kentucky, UNITED STATES

## Introduction

Macrodomain is a conserved protein fold, existing either as a single protein or embedded within a larger protein, that has been identified in viruses, bacteria, archaea, and eukaryotes (reviewed in [1–3]). Originally identified as X-domains in viruses [4], these conserved regions were renamed macrodomains due to their similarity to the C-terminal domain of the histone H2A variant called MacroH2A [5]. This protein domain typically consists of 130–190 amino acids that adopt a distinct fold consisting of a central beta sheet surrounded by 4 to 6 helices. Most macrodomains bind to monomeric ADP-ribose (MAR) and its derivatives, including ADP-ribose-1″-phosphate (Appr1p), O-acyl-ADP-ribose, and the terminal ADP-ribose of poly(ADP-ribose) (PAR), as well as protein-conjugated MAR or PAR (i.e., MARylated or PARylated proteins) [6–9]. A subset of macrodomains also possess enzymatic activity to hydrolyze these ADP-ribose derivatives. In this Pearl, we will use viruses as examples to discuss the significance of the macrodomain and its associated enzymatic activities in the pathogenesis of infectious diseases. The role of macrodomains from other pathogens will be briefly explored at the end.

## What does a macrodomain do?

**Fig 1:**
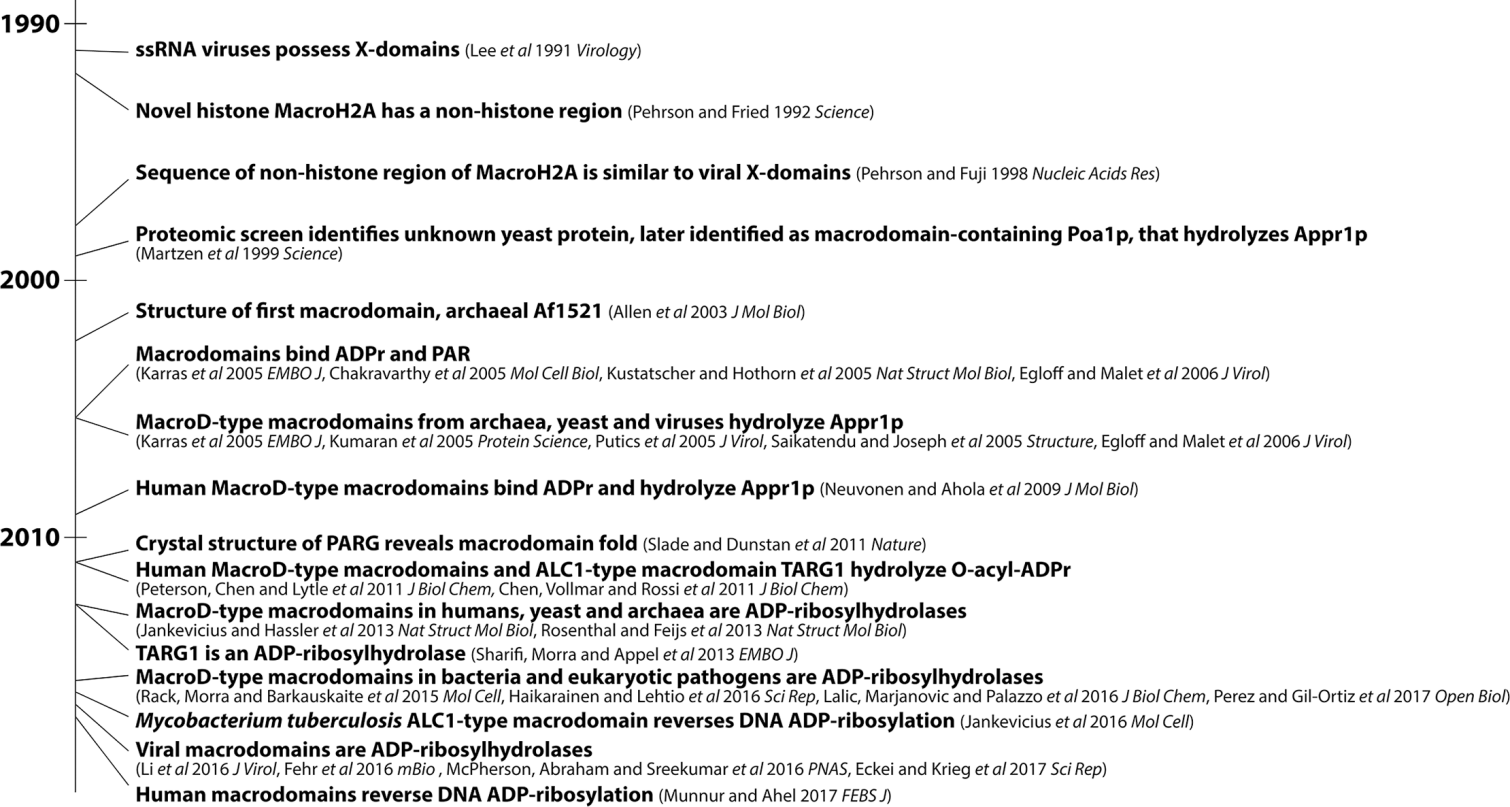
A brief timeline of the discovery of macrodomain functions. Significant advances in the macrodomain field starting from the initial identification of “X-domains” in coronaviruses by Lee et al. in 1991 [4] to the present. Findings are listed in bold followed by relevant citations.

Macrodomains were first associated with ADP-ribose through a yeast proteomics screen that identified an enzyme dephosphorylating Appr1p, a by-product of tRNA splicing [8] (a timeline of macrodomain discovery is presented in Fig 1). It was later demonstrated that the first structurally characterized macrodomain, archaeal *Af*1521, also possessed enzymatic activity towards Appr1p and specifically bound ADP-ribose, MARylated and PARylated proteins [6,9,10]. Subsequent studies demonstrated that some macrodomains, such as MacroH2A, possessed an affinity for free and protein-conjugated ADP-ribose but lacked hydrolysis activity [10–13]. Recent informatics analyses have revealed 6 macrodomain subclasses [1]. The MacroD subclass was identified in all kingdoms of life, including viruses [1], and this subclass was demonstrated in vitro to dephosphorylate Appr1p (e.g., [14]), deacetylate O-acyl-ADP-ribose [7], and hydrolyze ADP-ribose from MARylated proteins [12,13,15–18] and, in some cases, PARylated proteins [17,18] (the last activity against ADP-ribosylated proteins is termed ADP-ribosylhydrolase activity). However, the in vivo substrates of MacroD-type macrodomains in humans, viruses, and other species remain unclear.

## Are viral macrodomains important for virus replication and virulence?

**Fig 2:**
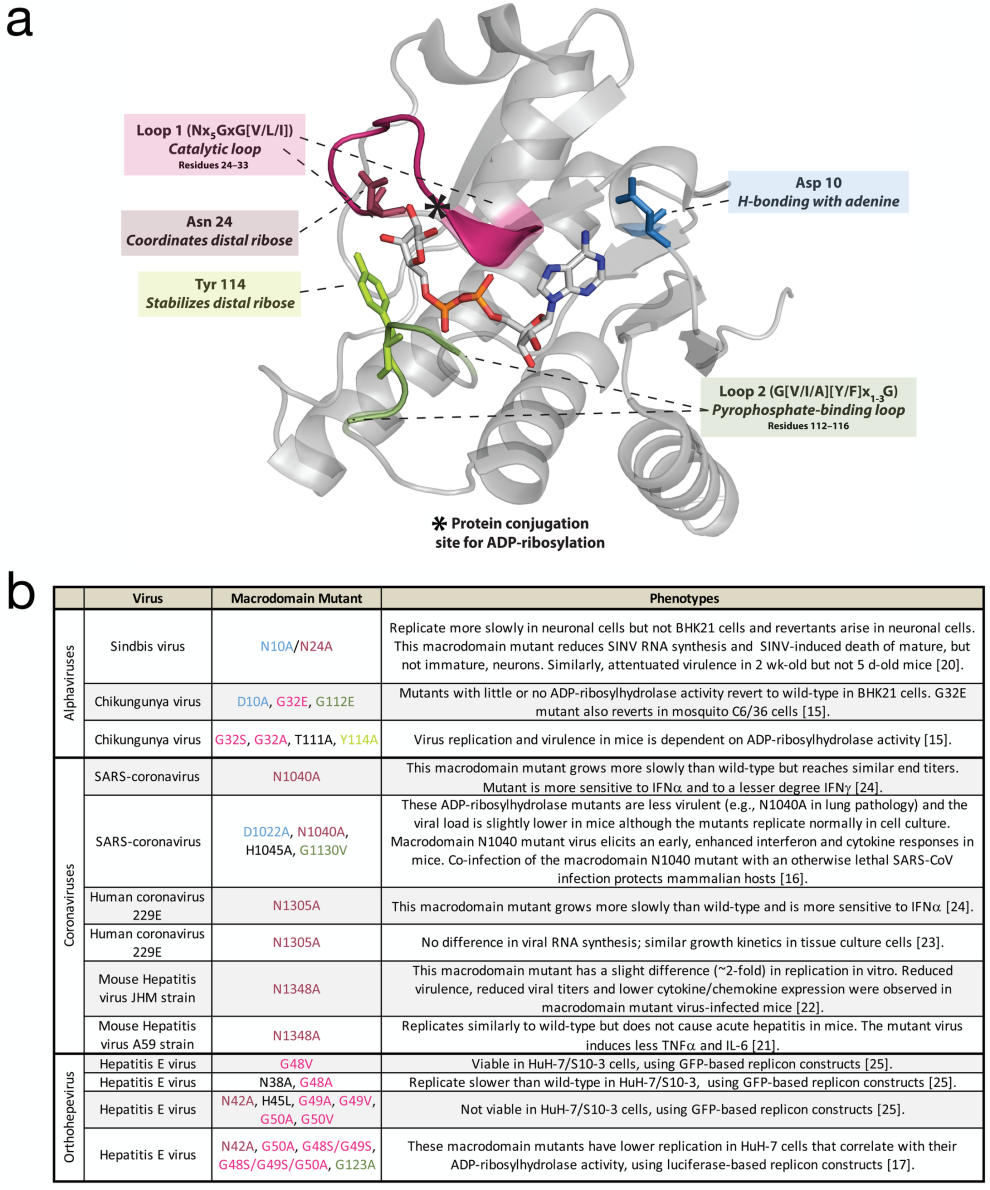
Structure and functions of viral macrodomains. **(A)** Ribbon representation of CHIKV nsP3 macrodomain (PDB: 3GPO, Malet et al. 2009 J Virol) in complex with ADP-ribose ligand. Conserved structural motifs critical for ligand recognition are highlighted in color and functionally conserved residues frequently mutated in studies of viral macrodomains are represented as sticks. **(B)** Phenotypes of macrodomain mutant viruses characterized in cells and in vivo. Corresponding residues and regions across different viruses are color-coded in panels a and b. ADP-ribose, adenosine diphosphate ribose; CHIKV, Chikungunya virus; PDB, Protein Data Bank.

MacroD-type macrodomains are present in the non-structural proteins of a subset of positive-strand RNA viruses, including alphaviruses, coronaviruses, rubella virus, and hepatitis E virus (HEV) [1,4]. The macrodomain sequence is highly conserved in the nsP3 non-structural protein of alphaviruses such as Sindbis virus (SINV) and Chikungunya virus (CHIKV) and coronaviruses such as the cause of severe acute respiratory syndrome (SARS). A role for nsP3 in virulence was first identified for the alphavirus Semliki Forest virus (SFV) [19]. Characterization of the functions of viral macrodomains has been aided by crystallography studies that identified critical residues for binding ADP-ribose (e.g., CHIKV structure in Fig 2A). In general, viruses with mutations in the ADP-ribose-binding sites of the macrodomain are not impaired for replication in most tissue culture cells, but often exhibit attenuated replication in differentiated cells and decreased virulence in vivo (Fig 2B) [16,20–25]. Comparable mutations in different classes of viruses often yield varying phenotypes. For example, mutations targeted at the ADP-ribose-binding site of the alphaviral macrodomain (SINV N10A, N24A) did not affect replication in baby hamster kidney (BHK21) cells but impaired replication in neurons and attenuated neurovirulence for mice [20]. Mutation of the coronavirus macrodomain at a site comparable to the alphavirus N24A site attenuated virulence and replication in mice and affected induction of and sensitivity to interferon (IFN) and inflammatory cytokines [16,21,22,24]. Mutation at the corresponding position in the HEV macrodomain results in reduced or no replication in liver cancer cell lines [17,25]. Biochemical studies have further identified residues responsible for enzymatic activities against various ADP-ribose derivatives [7,12,13,26,27]. Some mutations in the catalytic loop region in HEV and alphaviruses are not tolerated [15,25]. For example, G32E in CHIKV (Fig 2A, loop 1) rapidly reverts to the wild-type amino acid in both mammalian and mosquito cells that lack functional IFN responses, suggesting that the enzymatic activity may be critical for alphaviral replication in both the host and vector independent of an innate IFN response [15].

## Which in vivo substrates could viral macrodomains be targeting?

Different cellular pathways generate ADP-ribose derivatives in vivo: Appr1p is derived from tRNA splicing via tRNA phosphotransferase 1 (TRPT1) [28], O-acyl-ADP-ribose is a side product of NAD^+^-dependent deacetylation mediated via sirtuins (SIRT1-7) [29], and ADP-ribosylation is accomplished primarily by diphtheria toxin-like (ARTD) proteins, commonly known as poly(ADP-ribose) polymerases (PARPs) [30–32]. Given that mutations in the active site of viral macrodomains likely affect enzymatic activity toward all of these substrates, it is difficult to assign mutant phenotypes to specific ADP-ribose derivatives. Other criteria for in vivo macrodomain specificity should, therefore, be considered. For example, how conserved are macrodomain enzymatic activities across different viruses? How does the decreased virulence of mutants correlate with the deficiency of these activities? If the hydrolysis activity of viral macrodomains is involved in virulence, do the host enzymes that synthesize these ADP-ribose derivatives have antiviral properties?

Regarding enzymatic activities, Appr1p phosphatase activity is not conserved across different viruses (e.g., SFV possesses very poor activity), and the turnover rate of the enzyme is often low (k_cat_ = 5–20 min^−1^) [21–24,27,33]. While O-acyl-ADP-ribose deacetylase activity has yet to be determined for viral macrodomains, recent data indicate that macrodomains from all classes of viruses have robust ADP-ribosylhydrolase activity in vitro [15–18] and in cells [18]. Compared with Appr1p phosphatase activity, the ADP-ribosylhydrolase activity more consistently accounts for the in vivo phenotypes observed for mutants with disrupted activity. For example, the CHIKV macrodomain D10A mutant that possesses 50%–75% of Appr1p phosphatase activity but minimal ADP-ribosylhydrolase activity cannot be recovered, while the Y114A mutant with no phosphatase activity and approximately 40% ADP-ribosylhydrolase activity is viable [15,27].

Amongst these ADP-ribose derivatives, Appr1p is less likely to be a substrate for viral macrodomains in vertebrate hosts because it is generated through a 5′ phosphate ligation pathway of tRNA splicing that is common in yeast but not in vertebrates [28]. While all macrodomain-containing viruses replicate in the mammalian cell cytoplasm, most tRNA splicing occurs in the nucleus, whereas sirtuin-based deacetylation and ADP-ribosylation are present in both the cytoplasm and nucleus [28,29,34]. In humans, TRPT1 localizes in the mitochondria, and only 1 of 7 sirtuins localizes in the cytoplasm, whereas nearly all ADP-ribosyltransferases (except PARPs 1–3) localize to a significant extent in the cytoplasm [28,29,34]. Lastly, several PARPs, but not TRPT1 or any sirtuins, are induced by IFN as part of the vertebrate antiviral response [35]. Taken together, macrodomain ADP-ribosylhydrolase activity is likely critical for viral pathogenesis.

## What roles could PARPs play during viral infection?

Amongst the 17 PARPs in humans, 4 are capable of PARylation (PARPs 1, 2, 5a, and 5b), 11 add MARylation (PARPs 3, 4, 6, 7, 8, 10, 11, 12, 14, 15, and 16), and 2 are catalytically inactive (PARPs 9 and 13) [30–32]. Several pieces of evidence indicate that some PARPs may be critical for the host antiviral response. First, PARPs 4, 9, 13, 14, and 15 are under strong diversifying selection among mammals—an indicator of antiviral genes [36]. For PARPs 9, 14, and 15, the recurrent selection lies in their ADP-ribose-binding macrodomain, recently hypothesized to be “locked in antagonistic ‘arms races’ with viral factors” [36]. Second, ectopic expression of the alphaviral macrodomain-containing nsP3 protein inhibits the formation of stress granules—a class of cytoplasmic structures with antiviral functions (e.g., [37]; reviewed in [38]). Coincidentally, PARPs 5a, 12, 13, 14, and 15 are present in stress granules whose integrity is dependent on ADP-ribosylation [39]. Third, overexpression of PARPs 7, 10, or 12 inhibits alphavirus replication, suggesting that these PARPs have antiviral functions [40]. PARPs 9, 12, 13, and 14 are amongst the 62 IFN-stimulated genes conserved across vertebrates [35], underscoring the importance of ADP-ribosylation regulation in fighting virus infection.

**Fig 3:**
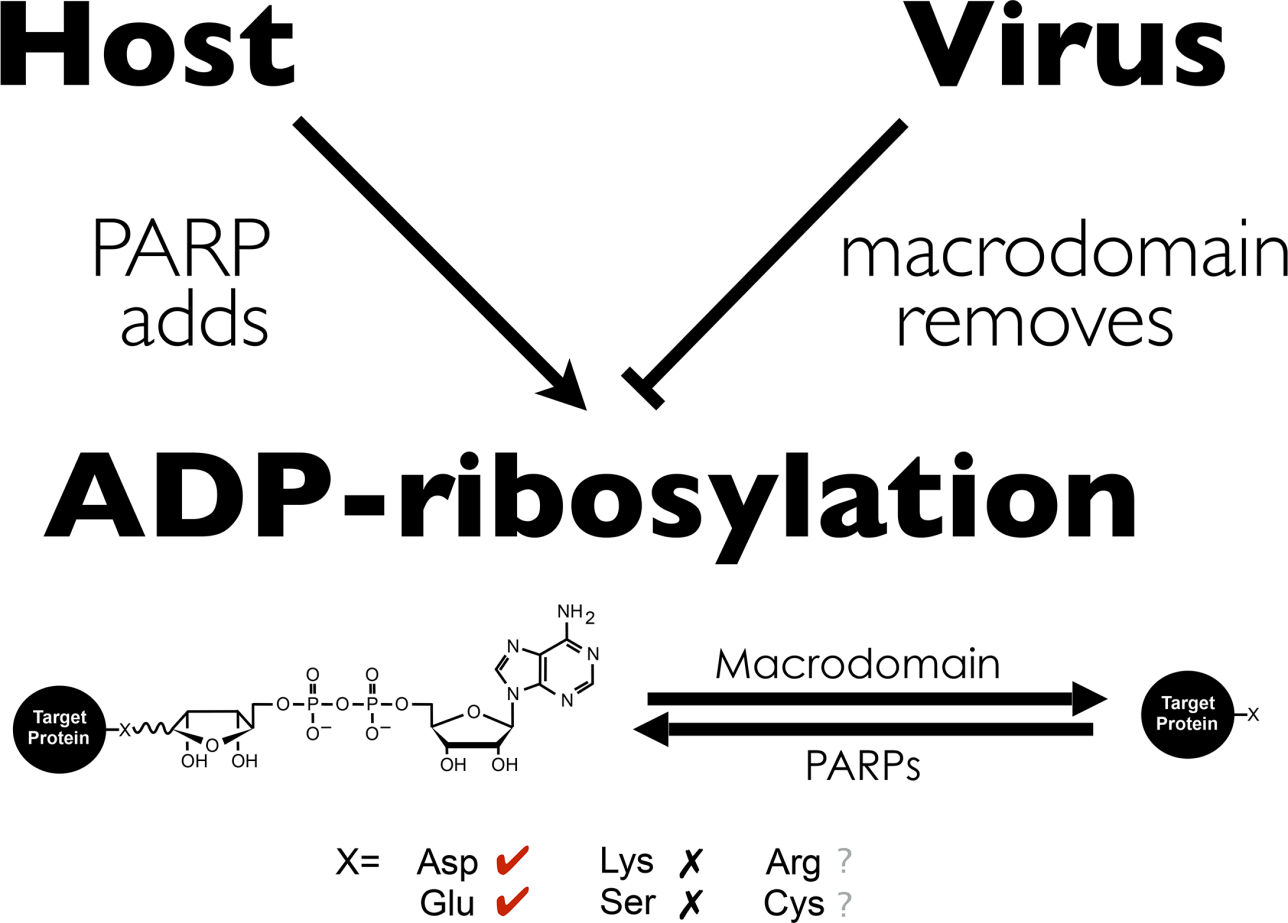
Model: ADP-ribosylation at the forefront of the battle between the virus and the host. A working model based on (1) ADP-ribosylation and host PARPs induced upon virus infection/interferon and (2) viral macrodomain possession of ADP-ribosylhydrolase activity. ADP-ribose can be conjugated to amino acids of diverse chemistry (Asp, Glu, Lys, Arg, Ser, and Cys). MacroD-type macrodomain, to which viral macrodomain belong, removes ADP-ribose conjugated to Asp and Glu, but not Lys and Ser. The ability of viral macrodomains to hydrolyze ADP-ribose from Arg and Cys remains unclear. ADP-ribose, adenosine diphosphate ribose; Arg, arginine; Asp, aspartic acid; Cys, cysteine; Glu, glutamic acid; Lys, lysine; PARP, poly(ADP-ribose) polymerase; Ser, serine.

One noteworthy feature of ADP-ribosylation is that though ADP-ribose can be conjugated onto a range of chemically diverse protein residues [41,42], MacroD-type macrodomains only have ADP-ribosylhydrolase activity for ADP-ribosylated aspartate and glutamate but not lysine or serine [12,13,15,43,44]. Moreover, most virus-induced PARPs add MARylation, while all viral macrodomains remove MARylation. Thus, one intriguing hypothesis is that while viral macrodomains can bind ADP-ribosylation added by host PARPs, viruses may circumvent host defenses or regulate replication by removing specific classes of ADP-ribosylation (Fig 3).

## Are there any additional interesting observations about macrodomains of RNA viruses and other pathogens?

Macrodomain variations exist between RNA viruses. For example, a helicase domain next to the MacroD-type macrodomain facilitates hydrolysis of PARylated substrates in HEV [17]. Coronaviruses possess tandem macrodomains following a MacroD-type macrodomain. These tandem domains do not bind ADP-ribose but instead bind nucleic acids [45], as do some MacroD-type viral macrodomains [1,14]. However, the physiological significance of nucleic acid binding of viral macrodomains remains unclear.

ADP-ribosylhydrolase activity has also been demonstrated in vitro for macrodomains from other pathogens, including *Trypanosoma brucei*, *T*. *cruzi*, *Staphylococcus aureus*, *Streptococcus pyogenes*, and *Streptomyces coelicolor* [46–48]. A recent study suggested that cross-talk between lipoylation and macrodomain-reversible ADP-ribosylation plays a vital role in regulating a pathogen's response to host-derived reactive oxygen species [46]. Therefore, it is possible that macrodomain ADP-ribosylhydrolase activity is critical for the pathogenesis of a broad spectrum of infectious diseases.

Although most macrodomains share a similarity in primary amino acid sequence, novel subclasses of macrodomains have been identified only by determining the 3-D structures [1–3]. Given the lack of conserved sequences between different macrodomain subclasses, it leaves open the possibility for more macrodomains and their functions to be discovered. *Mycobacterium tuberculosis* possesses a nonMacroD-type macrodomain, which removes ADP-ribosylation from DNA, rather than protein, and antagonizes the action of a mycobacterial toxin that ADP-ribosylates DNA at specific thymidines [49]. Lastly, besides the macrodomain family, there is another major class of ADP-ribosylation removal enzyme called DraG/ARH, and they are also found in all kingdoms of life, including viruses [42,50]. Unlike MacroD-type macrodomains, which remove ADP-ribosylation from acidic residues, ARH members remove ADP-ribosylation from arginine [51] and serine [43,52]. Therefore, one can speculate that pathogens may have multiple enzymes to remove ADP-ribosylation from specific classes of amino acids.
